# Production and Functional Evaluation of Anti-*Loxosceles* Sera Raised by Immunizations of Rabbits with Mutated Recombinant Phospholipases-D

**DOI:** 10.3390/biomedicines11010079

**Published:** 2022-12-28

**Authors:** Bruno Cesar Antunes, Nayanne Louise Costacurta Polli, Pedro Henrique de Caires Schluga, Thais Pereira da Silva, Ana Carolina Martins Wille, Rosangela Locatelli-Dittrich, Giovana Scuissiatto de Souza, Fernando Hitomi Matsubara, João Carlos Minozzo, Andrea Senff-Ribeiro, Luiza Helena Gremski, Silvio Sanches Veiga

**Affiliations:** 1 Department of Cell Biology, Federal University of Paraná (UFPR), Curitiba 81530-900, Brazil; 2 Production and Research Center of Immunobiological Products (CPPI), State Department of Health, Piraquara 83302-200, Brazil; 3 Department of Structural, Molecular Biology and Genetics, State University of Ponta Grossa (UEPG), Ponta Grossa 84030-900, Brazil; 4 Veterinary Hospital, Federal University of Paraná (UFPR), Curitiba 80035-050, Brazil

**Keywords:** brown spider, venom, loxoscelism, serum therapy, phospholipases D

## Abstract

Loxoscelism is the clinical condition triggered after the bite of spiders of the genus *Loxosceles*. The main species involved in accidents in South America are *L. intermedia*, *L. laeta,* and *L. gaucho*. The only specific treatment is the anti-*Loxosceles* serum produced with crude venoms. As phospholipases D (PLDs) trigger most of the effects observed in accidents, we developed and evaluated second-generation sera using mutated PLDs as antigens. Three isoforms of PLDs with site-directed mutations without biological activities were used for rabbit immunizations: D32A-E34A (*L. gaucho*), W230A (*L. intermedia*)*,* and H12A-H47A (*L. laeta*). Sera were produced using crude venoms of three species of *Loxosceles* enriched with mutated recombinant PLDs (MIX) or using only mutated PLDs (REC). Immunizations stimulated the immune system from the second immunization with higher antibody production in the REC group. In vivo neutralization assays demonstrated that both sera reduced edema and dermonecrosis caused by *Loxosceles intermedia* crude venom. Follow-up of animals during the immunization protocols and in the neutralization assays demonstrated that the mutated proteins and the sera are safe. Results demonstrate the potential of using mutated recombinant PLDs in total or partial replacement of *Loxosceles* venoms in animal immunizations to produce anti-*Loxosceles* sera for treatments of Loxoscelism.

## 1. Introduction

Accidents caused by bites of spiders of the genus *Loxosceles,* popularly known as Brown spiders, have been reported in different regions of the world [1,2]. In Brazil, it is considered that *Loxosceles intermedia, L. gaucho,* and *L. laeta,* found especially in the South and Southeast regions, are the species of greatest medical and epidemiological importance [1,3].

The clinical picture developed by patients bitten by Brown spiders is known as Loxoscelism [1] and can be classified into two variants named Cutaneous Loxoscelism, seen in most victims, and Systemic Loxoscelism, which has a lower incidence, but it is more severe [1,4]. In the cutaneous form, also designated as Dermonecrotic Loxoscelism or Gangrenous Arachnidism, some clinical signs can be observed at the bitten site and at the surrounding areas, such as edema, erythema, ecchymosis, formation of a marble plaque and dermonecrosis, with gravitational spreading [1,5,6]. On Systemic Loxoscelism, the presence of thrombocytopenia, hemoglobinuria, disseminated intravascular coagulation, intravascular hemolysis, and acute renal failure can be seen, which can lead to the death of some patients [1,5,6,7,8,9].

It is known that venom is a complex mixture of low molecular mass components (3–45 kDa) with toxic or enzymatic activities [1]. Identification and characterization studies of toxins present in *Loxosceles* venoms have revealed the presence of phospholipases D (30–35 kDa), astacins (metalloproteases) (20–35 kDa), knottins (Inhibitor Cystine Knot peptides) (3–10 kDa), hyaluronidases (41–43 kDa), serine proteases (85–95 kDa), allergen factor (42 kDa), TCTP (translationally controlled tumor protein) (22 kDa), and serpin (serine proteases inhibitor) (43 kDa) [10,11,12,13].

The best-characterized class of toxins corresponds to the phospholipases D (PLDs), which are also known as Dermonecrotic Toxins. Transcripts related to PLDs represent about 20% of toxin-encoding transcripts in the venom gland of *L. intermedia* [1,14]. These enzymes are highly conserved among different species of the genus and play a central role in the pathophysiology of envenoming [1,14]. The biological significance of PLDs can be seen in several studies that demonstrate that both the purified native PLDs from crude venoms, as well as their recombinant counterparts, could reproduce most of the toxic effects observed in accidents. PLDs target cell membranes and prompt the cleavage of phospholipids, releasing biochemical mediators. This molecular and cellular mechanism results in dermonecrosis and other systemic complications in the victims [14,15,16,17,18,19]. Through the description of the tridimensional structure of Brown spider venom PLDs using crystallography, the essential domains and amino acid residues involved in the enzymatic activity of these molecules were identified. In the catalytic site, it was described the presence of amino acid residues His 12 and His 47 conserved in all phospholipase D isoforms of *Loxosceles spp.* described to date. Other amino acid residues, such as Glu 32, Asp 34, and Asp 91, participate in the coordination of the magnesium ion, which is very important for enzyme activity. Additionally, the amino acid residues Y228, Y229, and W230 found in an exosite are related to the binding of the enzyme to its substrates (phospholipids) [1,14,20,21,22,23].

Through site-directed mutations in the wild-type PLDs, it was possible to obtain several soluble PLDs recombinant isoforms (H12A, H12A-H47A, E32A-D34A, W230A, Y228A-Y229A-W230A) of *L. intermedia, L. gaucho* and *L. laeta* venoms. Such mutations resulted in molecules without activities, incapable of degrading sphingomyelin and lysophosphatidylcholine or developing dermonecrosis in rabbits, in addition to the lack of hemolytic activity and no vascular permeability increase [22,24,25]. Considering that all mutated phospholipase D isoforms of the three Brown spider species studied maintained their conformational characteristics (determined by circular dichroism and docking analysis), as well as were recognized in ELISA and Western blotting studies by using anti-*Loxosceles* crude venoms and anti-wild-type recombinant PLD antibodies [22,24,25], the possibility of using these antigens on immunization protocols to produce a new serum for the treatment of loxoscelism was raised.

Although there are some difficulties in diagnosing accidents caused by *Loxosceles* spiders, especially in the first hours, and considering that there is certain heterogeneity in the approach and dispensing of drugs to injured patients [26], it is known that serum therapy with anti-*Loxosceles* serum is the available methodology with the greatest therapeutic potential, since it has specific antibodies capable of neutralizing Brown spider venom toxins [27]. However, the production of anti-*Loxosceles* serum using crude venoms has critical points that can even be considered limiting to the manufacture of the product, such as the need to capture thousands of spiders from the natural environment, ex situ maintenance under laboratory conditions and venom extraction of a great number of spiders, which makes the work risky, laborious, and costly, in addition to the low yield in the venom extractions. Besides that, the need to immunize horses with crude venom, which potentially causes damage and suffering to animals, is another critical point regarding the production chain. In this sense, some research groups have been working in recent years to obtain recombinant molecules without biological activities but with preserved conformational characteristics and immunogenic properties, which can be used in immunization protocols aiming at the development of a new generation serum [24,28,29,30].

Therefore, we present in this work the production of a new anti-*Loxosceles* toxin serum produced in rabbits immunized with mutated isoforms of PLDs present in the *Loxosceles intermedia* (LiRecDT1 W230A), *L. laeta* (LlRecDT1 H12A-H47A) and *L. gaucho* (LgRecDT1 D32A-E34A) venoms. These isoforms were chosen to be applied as antigens because each one contained a different mutation and rendered an antigen preparation with PLDs from the three species. Results concerning the pre-clinical neutralizing capacity and analysis of possible toxic effects caused during their use as antigens to immunization protocols are also shown.

## 2. Materials and Methods

### 2.1. Preparation of Venom Solutions

The solutions of *L. intermedia*, *L. laeta,* and *L. gaucho* venoms were prepared and provided by CPPI (Production and Research Center of Immunobiological Products, State Department of Health, Piraquara, Paraná, Brazil). Adult spiders’ specimens were collected from natural areas in the states of Paraná and Santa Catarina (authorization numbers 028/2021—PR and 002/2021—SC) and maintained at CPPI. Venoms were extracted by electrostimulation (15 V) applied to the spiders’ cephalothorax near the chelicerae, followed by collection with a micropipette. After that, the venom’s samples were crystallized, diluted in 0.85% saline, filtered (0.22 µm), and maintained at −20 °C until use. The final protein contents of venom’s solutions were determined by the Coomassie blue method (Bio-Rad, Hercules, CA, USA), as described by Bradford [31].

### 2.2. Animals

Adult New Zealand rabbits (~2.5 kg) were acquired from the Canguiri Experimental Farm of the Federal University of Paraná and randomly selected for immunization protocols and in vivo experiments. All procedures involving animals were carried out in accordance with Brazilian Federal laws, following the Institutional Ethics Committee for Animal Studies Guidelines from the Federal University of Paraná (certificate no. 1238).

### 2.3. Production and Characterization of Recombinant Phospholipases D (PLDs)

#### 2.3.1. Recombinant Expression and Purification

Wild-type PLDs designated as LiRecDT1 (from *L. intermedia*), LlRecDT1 (from *L. laeta*), and LgRecDT1 (from *L. gaucho*) [16,24], and PLDs with site-directed mutations designated as LlRecDT1 H12A-H47A (from *L. laeta*), LgRecDT1 E32A-D34A (from *L. gaucho*) and LiRecDT1 W230A (from *L. intermedia*) [22,24] were expressed as described next. Wild-type and mutated constructions based on pET-14b vectors were transformed into *Escherichia coli* BL21(DE3)pLysS, which were plated onto Luria-Bertani (LB) agar plates containing 100 µg/mL of ampicillin and 34 µg/mL of chloramphenicol. Single colonies of PLDs were used to inoculate 10 mL of liquid LB broth containing the antibiotics specified and grown overnight (16 h) at 37 °C, followed by dilution of this culture in 1 L of liquid LB plus antibiotics. The culture was allowed to grow at 37 °C under agitation (180 rpm) and monitored until the optical density (550 nm) reached between 0.4 and 0.6. The inducer (isopropyl β-D-thiogalactoside) was then added to a final concentration of 0.05 mM, and the culture was incubated at 30 °C for an additional 3.5 h under vigorous shaking. Subsequently, cells were harvested and resuspended in binding buffer (50 mM NaH_2_PO4/Na_2_HPO_4_ pH 8.0, 500 mM NaCl, 10 mM imidazole) under gentle agitation. Lysozyme was then added to a final concentration of 1 mg/mL, and this suspension was stored at −20 °C. After at least 16 h, the suspension was thawed, submitted to ultrasonic cell lysis, and centrifuged (9000× *g*, 30 min at 4 °C). The resultant supernatant was then filtered (0.22 µM) and subjected to the purification of the recombinant PLDs using a HisTrap™ HP 1 mL column coupled to an ÄKTA™ pure chromatography system (GE Healthcare Life Sciences). Column equilibration and washing steps were performed with washing buffer (20 mM NaH_2_PO_4_, 500 mM NaCl, 20 mM imidazole, pH 7.5), and elution was executed with a linear gradient of elution buffer (20 mM NaH_2_PO_4_, 500 mM NaCl, 500 mM imidazole, pH 7.5). Purified PLDs were dialyzed against Phosphate-Buffered Saline (PBS) (100 mM NaCl, 80 mM Na_2_HPO_4_, 20 mM NaH_2_PO_4_, pH 7.4) and analyzed using a 12.5% SDS-PAGE under reduction conditions.

#### 2.3.2. Sphingomyelinase Activity

The enzymatic activity of wild-type and mutated recombinant PLDs was quantified using the Amplex™ Red Phospholipase D Assay Kit (Thermo Fisher Scientific, Waltham, MA, USA). Ten micrograms of mutated PLDs and the wild-type proteins LlRecDT1 and LgReDT1 were incubated with the Amplex™ Red reagent and sphingomyelin mixture in reaction buffer (Tris-HCl 100 mM pH 7.4 containing 10 mM MgCl_2_) for 30 min at 37 °C. After incubation, fluorescence was determined on a fluorimeter (Tecan Infinite M200, Männedorf, Switzerland) using excitation wavelength at 540 nm and emission at 570 nm [18].

#### 2.3.3. In Vivo Dermonecrosis

Mutated toxins (50 µg/each) diluted in PBS were intradermally injected into a shaved area of the rabbits’ skin (*n* = 3). Wild-type recombinant isoform LiRecDT1 (10 µg) was used as the positive control, and PBS represented the negative control. The injection areas were observed and photographed at 0, 3, 6, and 24 h post-injection. Signs of edema, bruise, and necrosis were analyzed and documented [22].

### 2.4. Production of Sera and Neutralization Assays

#### 2.4.1. Immunization Protocols

Twenty New Zealand rabbits were divided into four groups and used to produce the sera, as described in Table 1. Large amounts of recombinant proteins were used as they presented no biological activity in initial tests (results presented here and in [25]). In contrast, low amounts of venom were used as antigens based on the known toxic activity of whole *Loxosceles* venoms. Immunizations were performed at intervals between two and three weeks in the first and second immunizations and intervals between four and six weeks in subsequent immunizations. Injections were divided into three subcutaneous (s.c.) injection sites per animal [32]. Samples of 60 mL of blood were collected 15 days after the last injection and processed [32] to produce hyperimmune sera.

#### 2.4.2. Hematological and Biochemical Parameters

Hematological and biochemical parameters of blood samples collected from the rabbits used to produce the sera were analyzed to verify eventual toxicity derived from the immunization. For this, the samples were collected before each immunization step and submitted to different analyses. Red and white blood cell counts, hemoglobin concentration, and red blood cell indices were determined by using a hematology analyzer (Mindray Headquarters, Shenzhen, China) according to [33]. Biochemical parameters included aspartate aminotransferase, creatinine, muscular creatine kinase, urea, total plasma protein, and fibrinogen; these parameters were quantified by means of commercial kits from Bioclin (Belo Horizonte, MG, Brazil) applied in BS-200 Chemistry Analyzer (Mindray Headquarters, Shenzhen, China) and according to [34]. The statistical analyses were performed by two-way ANOVA with Tukey’s post-test, and the significance was established at *p* < 0.05 using the software GraphPad Prism 8.0.2.

#### 2.4.3. Clinical Follow-Up of Immunized Rabbits

Various parameters such as body weight, rectal temperature, edema, erythema, ecchymosis, and necrosis formation were checked from the time of immunization through the next seven days. Erythema and ecchymosis were classified from 1 to 4 according to their intensity, in which “1” represents a less intense reaction and “4” indicates a more intense sign (adapted from [35]). The injection area with the presence of a lesion or with a more intense lesion was used for this analysis. Statistical analyses were performed using a two-way analysis of variance (ANOVA) followed by a post hoc Tukey test.

#### 2.4.4. Immunoassays

ELISA assays were adapted from [36] and performed to evaluate the production of antibodies during the immunization process, as well as the profile of conformational epitopes recognized. Briefly, 96 well MaxiSorp plates (Nunc—ThermoFisher, Waltham, MA, USA) were coated with 600 ng of *L. intermedia* venom diluted in bicarbonate buffer (NaHCO_3_ 0.02 M pH 9.6). After washing with saline containing 0.05% of Tween 20, the plates were blocked with 2% powdered casein-PBS for 1 h at 37 °C. Sera from immunized rabbits were then added at 1:400 dilutions and incubated at 37 °C for 1 h. To exclude nonspecific reactions, pre-immune sera and controls without primary antibodies or antigens were also tested. After the washing step, the plates were incubated for 1 h at 37 °C with anti-rabbit IgG conjugated with horseradish peroxidase (Sigma-Aldrich A0545, Saint Louis, MO, USA) diluted 1:5000. Plates were washed again, and colorimetric reactions were carried out in the absence of light for 30 min at 25 °C using 0.4 mg/mL of o-phenylenediamine and 4 μL/mL H_2_O_2_ in citrate buffer (50 mM NaH_2_PO_4_, 24 mM sodium citrate, pH 5.0). The reactions were stopped by adding 20 μL of 1 M H_2_SO_4_ per well. Absorbance values were measured on a plate reader (Meridian ELX 800) at 490 nm. Samples were analyzed in triplicates, and statistical analyses were performed using a two-way analysis of variance (ANOVA) followed by a post hoc Tukey test. The significance was established at *p* < 0.05 using the software GraphPad Prism 8.0.2 (San Diego, CA, USA).

For the Western blotting experiments, 10 µg of venom samples was analyzed in SDS-PAGE 12.5% under reducing conditions and transferred onto nitrocellulose membranes. The membranes were then incubated with the produced rabbit sera diluted 1:1000. Alkaline phosphatase-coupled anti-rabbit IgGs (1:5000) (Sigma-Aldrich A3812, Saint Louis, MO, USA) was used as secondary antibodies, and the reaction was developed with BCIP/NBT (Promega) as substrate.

#### 2.4.5. Neutralization Assays

Samples of 3.05 µg of *L. intermedia* venom corresponding to 1 MND (Minimum Necrotizing Dose, which is the amount of venom that induces a lesion of approximately 1 cm^2^) were injected in the ear pinna of rabbits (*n* = 5). Right after the venom injection, 1 mL of the tested serum was administered via the marginal ear vein of the opposite ear. After 0, 24, 72, and 240 h of serum inoculation, the lesions were photographed, and the diameters of the dermonecrotic lesions at the venom injection sites were measured with a Vernier caliper to determine the area. Additionally, the body temperature of the rabbits was measured, and blood samples were collected from the central ear vein to verify the hematological and biochemical parameters described in Section 2.4.2 and Section 2.4.3.

Alternatively, samples of 3.05 µg (1 MND) or 6.1 µg (2 MND) of *L. intermedia* venom were incubated with each serum (total volume: 100 µL) for 1 h at 37 °C, and the mixture was injected (i.d.) in a back shaved area of rabbits’ skin. Animals were evaluated after 0, 24, 72, and 240 h of injections when the diameters of dermonecrotic lesions and edema near the inoculation sites were measured with a Vernier caliper to determine the areas. Erythema and ecchymosis were classified according to their intensity, as described in Section 2.4.3.

#### 2.4.6. Histological Analysis

Skin samples from rabbits exposed for 48 h to the incubation mixture of 2 MND of *L. intermedia* venom plus different sera were fixed in ALFAC solution (ethanol 85%, formaldehyde 10%, and glacial acetic acid 5%) at 4 °C for 16 h. After, the samples were dehydrated in a graded series of ethanol and embedded in paraffin for 2 h at 58 °C. Materials were then cut into thin sections (4 μm) and stained with Hematoxylin and Eosin. Images were observed under a light microscope Olympus BX41 and photographed with a DP 72 camera (Olympus, Tokyo, Japan).

## 3. Results

### 3.1. Mutated Recombinant PLDs Are Devoid of Enzymatic and Dermonecrotic Activities

**Figure 1:**
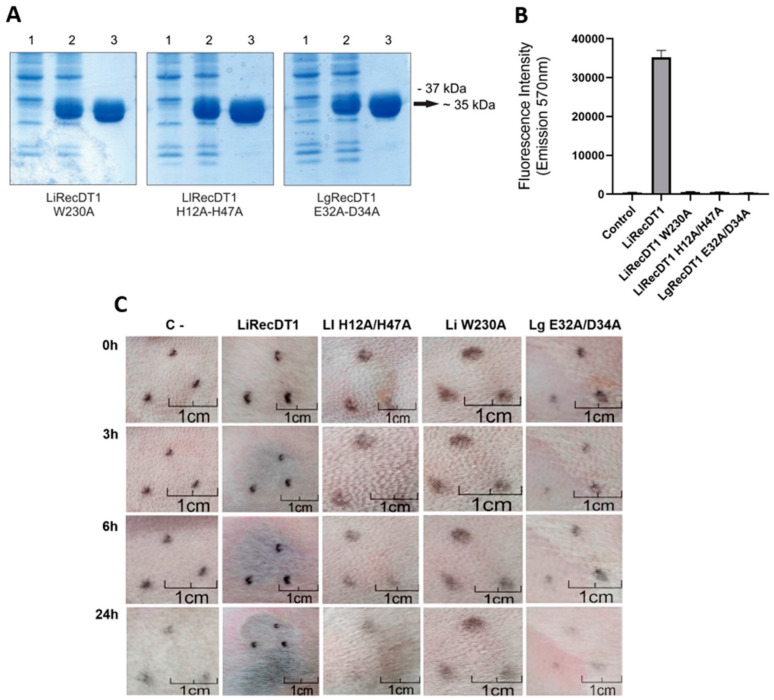
Properties of mutant phospholipases D (PLDs). (**A**) Recombinant expression and purification of PLDs analyzed by 12.5% SDS-PAGE under reducing conditions and stained with Coomassie blue dye. Lanes 1 show *E. coli* BL21(DE3)pLysS culture collected by centrifugation and resuspended in Laemmli sample buffer prior to induction with IPTG. Lanes 2 show the supernatant of cell lysates after induction with IPTG. Lanes 3 show purified eluted recombinant proteins (5 µg) through Ni^2+^-NTA agarose column. The position of the closest molecular mass standard and an arrow indicating the recombinant PLDs are depicted on the right of the figure. (**B**) Sphingomyelinase D activity of wild type (LiRecDT1) and mutated (LiRecDT1 W230A, LlRecDT1 H12A-H47A, and LgRecDT1 E32A-D34A) PLDs evaluated by Amplex Red Phospholipase D Assay Kit. The product of reaction was measured on a fluorimeter using excitation wavelength at 540 nm and emission at 570 nm. Control reaction was performed without any toxin. Values represent average ± SD of three independent experiments carried out in triplicate. (**C**) Macroscopic analysis of rabbit skin exposed to wild-type (10 µg) and mutated (50 µg) PLDs. Photographs show the injection area immediately after inoculation (0 h) and after 3, 6, and 24 h following injection. C-: Injection of PBS. Black dots marked on the skin surround the local injection.

PLDs from different species belonging to the genus *Loxosceles* generally share key amino acid residues for catalysis and binding to substrates. In order to produce inactive toxins that keep the three-dimensional conformation of wild-type PLDs, refs [22,24] performed site-directed mutagenesis protocols targeting some of these PLDs’ amino acids (H12, H47, E32, E34, and W230) from three *Loxosceles* species, and generated the constructions LlRecDT1 H12A-H47A (from *L. laeta*), LgRecDT1 E32A-D34A (from *L. gaucho*), and LiRecDT1 W230A (from *L. intermedia*). Here, these constructions were expressed in *E. coli* cells from BL21(DE3)pLysS strain and purified by affinity chromatography. As observed in Figure 1A, all recombinant isoforms were produced and purified as soluble proteins and appeared as ~35 kDa polypeptides in the 12.5% reducing SDS polyacrylamide gel. The sphingomyelinase activity of the recombinant proteins was quantified by the Amplex™ Red Phospholipase D Assay Kit, and results showed that all mutated isoforms have no enzymatic activity on sphingomyelin, while such activity is highly detected when the wild-type isoform LiRecDT1 is tested (Figure 1B). These enzymatic profiles observed for the isoforms corroborate with their in vivo activity observed after 24 h of injection in the skin of rabbits (Figure 1C). The macroscopic lesion observed near the injection site of the wild-type isoform LiRecDT1 displays the usual signs triggered by *Loxosceles* PLDs, such as ecchymosis, erythema, necrosis, and gravitational spreading of the lesion. Conversely, it is possible to observe that the mutated isoforms did not trigger any of these signs, as the macroscopic aspect of the injection area is quite similar to what was observed for the negative control (Figure 1C).

### 3.2. Sera Production: Effects of Immunizations

**Figure 2:**
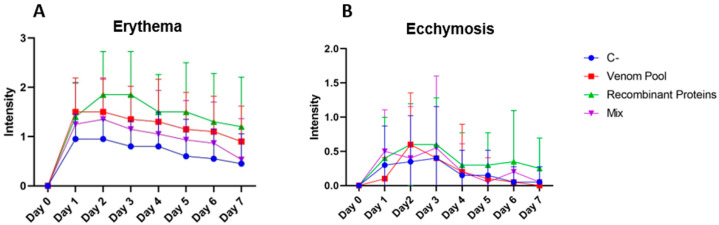
Monitoring of antigen injection sites of immunized rabbits. Erythema (**A**) and ecchymosis (**B**) were checked for seven days (X axis) following the immunizations with different antigens. Venom pool: pool of *L. intermedia*, *L. laeta* and *L. gaucho* venoms + adjuvant. Recombinant proteins: pool of mutated PLDs + adjuvant. Mix: venom pool + recombinant protein’s pool + adjuvant. C-: saline + adjuvant. The intensity of erythema and ecchymosis (Y axis) were classified from 1 to 4 according to the intensity, in which “1” represents a less intense reaction and “4” indicates a more intense sign (adapted from [35]). Each point represents the averages of intensities measured after immunizations. For erythema, results were statistically significant for Venom pool, Recombinant proteins (ANOVA, *p* < 0.0001 for both), and Mix (ANOVA, *p* = 0.0111) compared with C- group.

For the sera production, rabbits were immunized with four antigen preparations as previously described: (1) whole venom of *L. intermedia*, *L. laeta,* and *L. gaucho*, and this group was named AV (derived from antivenom); (2) recombinant mutated proteins LlRecDT1 H12A-H47A, LgRecDT1 E32A-D34A and LiRecDT1 W230A, which was named REC (derived from recombinant); (3) both the venom of the three *Loxosceles* species and the three recombinant mutated proteins, and this group was named MIX.; (4) PBS (with adjuvant), which was named C- (derived from negative control). Macroscopic and clinical signs were monitored during the seven days following all immunizations. One of the macroscopic signs observed after immunizations was local edema, and it was generally detectable one day after the injections. All animals administered with the antigens—whole venom, recombinant proteins, or both antigens (mix)—developed edema in at least one site of injection, while 80% of rabbits (*n* = 4) from the C- group showed this sign. This reaction was triggered after immunizations with either CFA (Complete Freund’s Adjuvant) or IFA (Incomplete Freund’s Adjuvant). Erythema was also observed after immunization in all animals from all groups; however, the intensity of erythema in the C- group was significantly lower than that from the three other groups that received the specific antigens (Figure 2A). In addition, the intensity of erythema detected in the animals immunized with the recombinant proteins (REC) was significantly higher than that from the other groups. Ecchymosis was also detected in all groups but not in all immunizations. This sign was mostly observed between the first and third days after injection and was significantly more intense in the animals from the REC group when compared to C- and AV (Figure 2B). All these signs—edema, erythema, and ecchymosis—progressively decreased in the days following the immunizations. Necrosis resulting from the immunizations was not observed in any of the treatments. The rectal temperature of immunized rabbits was the clinical sign checked in the days following immunizations. Results indicate that none of the injections induced fever, as the average temperatures of all animals did not exceed 40 °C during all experiments. For rabbits, the body temperature is considered normal when it is below 40 °C.

**Figure 3:**
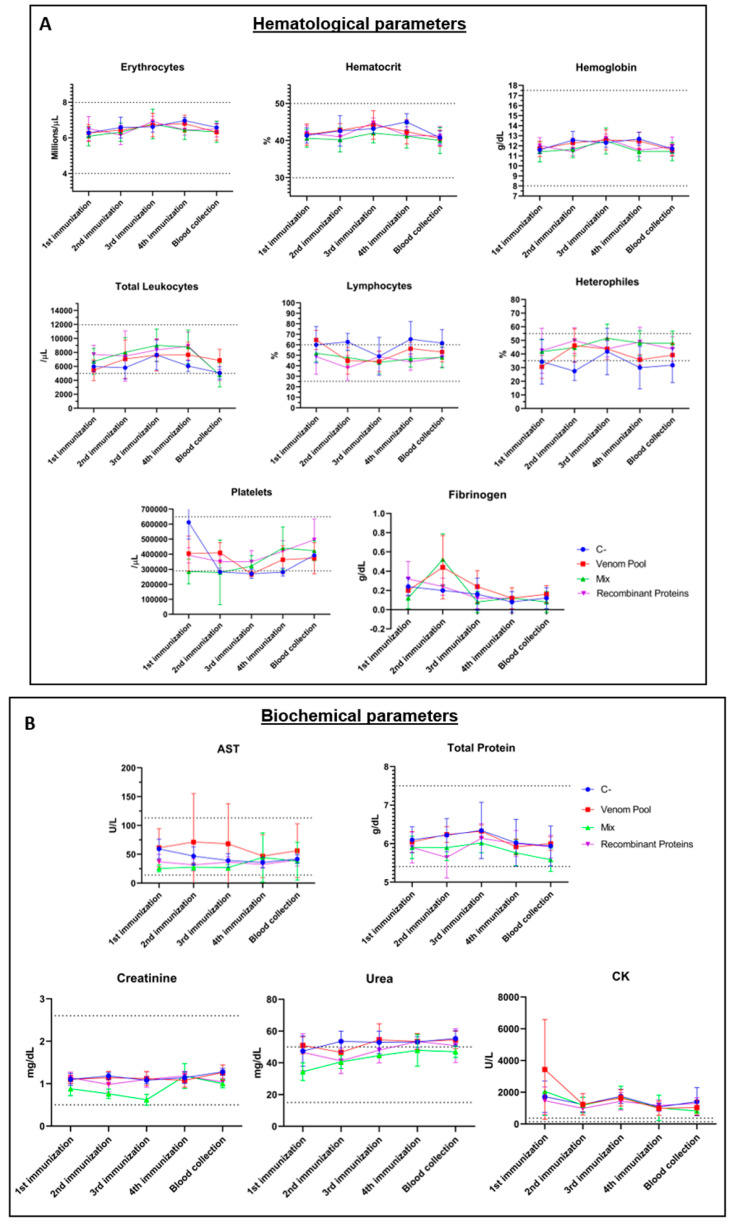
Monitoring of blood parameters of immunized rabbits. Hematological (**A**) and biochemical (**B**) parameters were verified from blood samples collected immediately before each immunization step. Venom pool: pool of *L. intermedia*, *L. laeta,* and *L. gaucho* venoms + Complete Freund’s Adjuvant (CFA) in the 1st immunization and Incomplete Freund’s Adjuvant (IFA) in the following immunizations. Recombinant proteins: pool of mutated PLDs + CFA in the 1st immunization and IFA in the following immunizations. Mix: venom pool + a recombinant protein’s pool + CFA in the 1st immunization and IFA in the following immunizations. C-: saline + CFA in the 1st immunization and IFA in the following immunizations. AST: aspartate aminotransferase. CK: muscular creatine kinase. Lymphocytes: relative lymphocyte count. Heterophiles: relative heterophile count. The dashed lines indicate upper and lower reference values. For the relative lymphocyte count, results were statistically significant for Recombinant proteins (ANOVA, *p* = 0.0002) and Mix (ANOVA, *p* = 0.0015) compared with C- group. For the relative heterophile count, results were statistically significant for Recombinant proteins (ANOVA, *p* = 0.0006) and Mix (ANOVA, *p* = 0.0002) compared with C- group. For AST, results were statistically significant for Recombinant proteins (ANOVA, *p* = 0.0355) and Mix (ANOVA, *p* = 0.0141) compared with Venom pool group. For Total protein, results were statistically significant for Mix compared with C- group (ANOVA, *p* = 0.0428). For creatinine, results were statistically significant for Mix compared with the other groups (ANOVA, *p* < 0.0001). For Urea, results were statistically significant for C- (ANOVA, *p* < 0.0001) and Venom pool (ANOVA, *p* = 0.0002) compared with Mix group.

Hematological and biochemical parameters were also monitored in blood samples collected right before all immunizations and fifteen days after the last treatment injection. Red blood cell count, hematocrit, and hemoglobin concentration remained within the reference values throughout the immunization process, in all groups, with no significant differences among them (Figure 3A). Serum fibrinogen levels also remained normal during the process in all groups and with no significant differences among them, although it is possible to observe a slight increase in fibrinogen levels in the AV and MIX groups after the first immunization (Figure 3A). In addition, no difference among groups was observed regarding the platelets’ count, although the C- group presented a close to the limit decrease between the second and fourth immunizations, which returned to normal values at the end of the process (Figure 3A). The hematological parameters concerning white blood cells reveal that the total leukocyte count did not vary significantly among groups during the immunization process. However, this leukocyte count decreased in all groups at the end of the protocol, especially in the MIX group, in which, at this point, a leukocyte count below the reference values was observed (Figure 3A). On the other hand, relative lymphocyte and heterophil counts revealed significant differences among groups. Before the second and fourth immunizations, the C- group statistically differed from the MIX and REC groups (Figure 3A). These differences are due to the high counts of lymphocytes in the C- animals, which balances with the low counts of heterophiles in the same group. Among the biochemical parameters evaluated during the process (Figure 3B), creatinine kinase (CK) from all groups and urea from C- and MIX groups (mainly after the third immunization) presented values above the limits for rabbits. Significant differences can be observed among some groups at certain steps of the treatment process (Figure 3B). However, as little variation exceeding the reference values was observed, no important physiological outcomes can be inferred.

### 3.3. Evaluation of the Produced Sera

**Figure 4:**
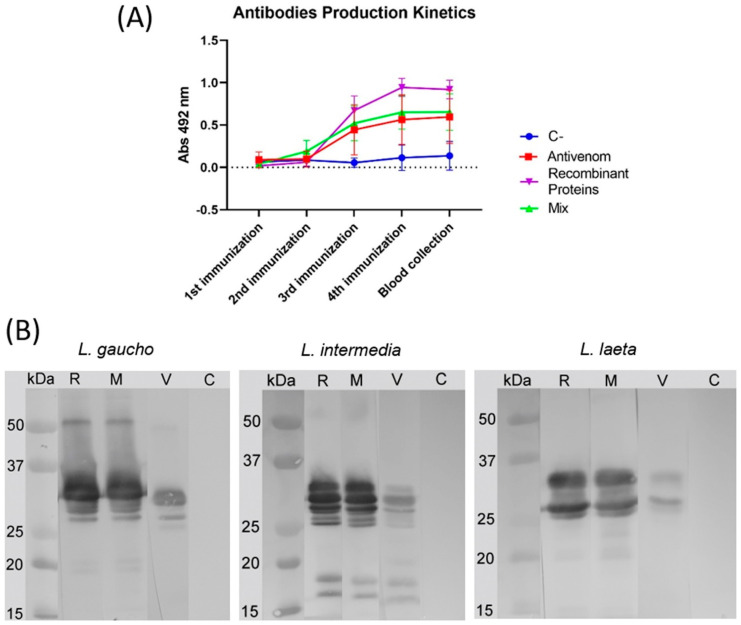
Reaction of produced sera with *Loxosceles* venoms. (**A**) ELISA for antibody capture assay with plates coated with *L. intermedia* venom (600 ng/well) and reacted with sera collected immediately before each immunization step (1:400 dilution). Antivenom: AV serum. Recombinant proteins: REC serum. Mix: MIX serum. C-: C serum. A pre-immune serum was used as control. Samples were analyzed in triplicates, and each point represents the mean ± SD. Results were statistically significant for all groups compared with C- (ANOVA, *p* < 0.0001). Results of Recombinant proteins’ group were statistically different from all other groups (ANOVA, *p* < 0.0001). No significant difference was found between Antivenom and Mix groups. (**B**) Western blotting showing immunological cross-reactivity among *Loxosceles* venoms and produced sera. Samples of venom (10 µg) from *L. gaucho*, *L. intermedia,* and *L. laeta* were probed with the produced sera (1:1000 dilution). R: REC serum. M: MIX serum. V: AV serum. C: C serum. A pre-immune serum was also used as control. Molecular protein mass standards are shown on the left of figures.

The production of antibodies resulting from the immunization process and their recognition profile were analyzed in vitro using ELISA and Western blotting analyses. The four produced sera (AV, REC, MIX, and C) were evaluated and compared during the immunization protocol, as blood samples of all animals were collected right before each immunization until the bleeding for sera production (blood collection). First, an ELISA was performed to quantify the recognition of native epitopes (conformational and linear) from venom antigens by the produced sera. As depicted in Figure 4A, there was an increase in the production of specific antibodies after the second immunization in the AV, REC, and MIX groups, with a tendency for curves to grow until the fourth inoculation and stabilization of the antibody’s levels when the bleeding step for sera production was performed. After the second immunization, all groups immunized with specific antigens presented a significantly higher production of antibodies when compared to the C- group. Furthermore, REC sera showed a higher response when compared to AV and MIX groups. Finally, Western blotting analysis was performed to verify the recognition profile of linear/denaturation-resistant epitopes from venom proteins by the produced sera. As displayed in Figure 4B, all specific sera recognized the proteins of different *Loxosceles* venoms with molecular weights between 25 and 35 kDa. Nevertheless, it is possible to observe that the bands produced by AV serum are less intense than those from REC and MIX sera.

### 3.4. Neutralization of In Vivo Toxicity

**Figure 5:**
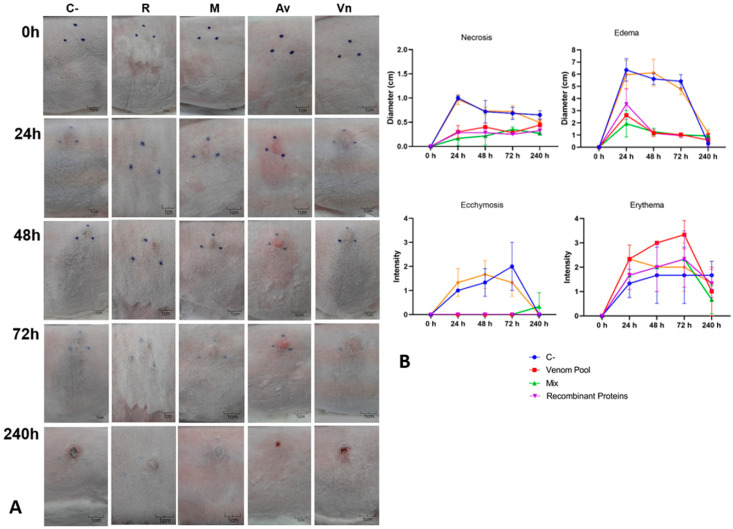
Neutralization of cutaneous loxoscelism triggered by 1 MND of venom tested with pre-incubation. One MND (3.05 µg) of *L. intermedia* venom previously incubated with each serum was injected (I.D.) in the back skin of rabbits (groups of five animals). (**A**) Macroscopic aspect of injected area after the indicated time courses. C-: C serum. R: REC serum. M: MIX serum. Av: AV serum. Vn: 1 MND of *L. intermedia* venom in the absence of serum. Photographs show a representative response to the animals. Black dots marked on the skin surround the local injection. (**B**) Skin necrosis, edema, ecchymosis, and erythema were checked after various time courses (X axis) following injections of 1 MND of venom pre-incubated with each serum. The intensity of such signs (Y axis) was classified from 1 to 4 according to the intensity, in which “1” represents a less intense reaction and “4” indicates a more intense sign (adapted from [35]). Each point represents the average intensities of each group measured at the indicated times ± SD. Antivenom: AV serum. Recombinant proteins: REC serum; Mix: MIX serum. C-: C serum. *L. intermedia* venom: 1 MND of *L. intermedia* venom in the absence of serum. For necrosis, edema, and ecchymosis, results of the groups AV, MIX, and REC were statistically different from C (ANOVA, *p* < 0.0001). No significant difference was found among groups when erythema was analyzed. In all analyses, no significant difference was found among AV, MIX, and REC.

**Figure 6:**
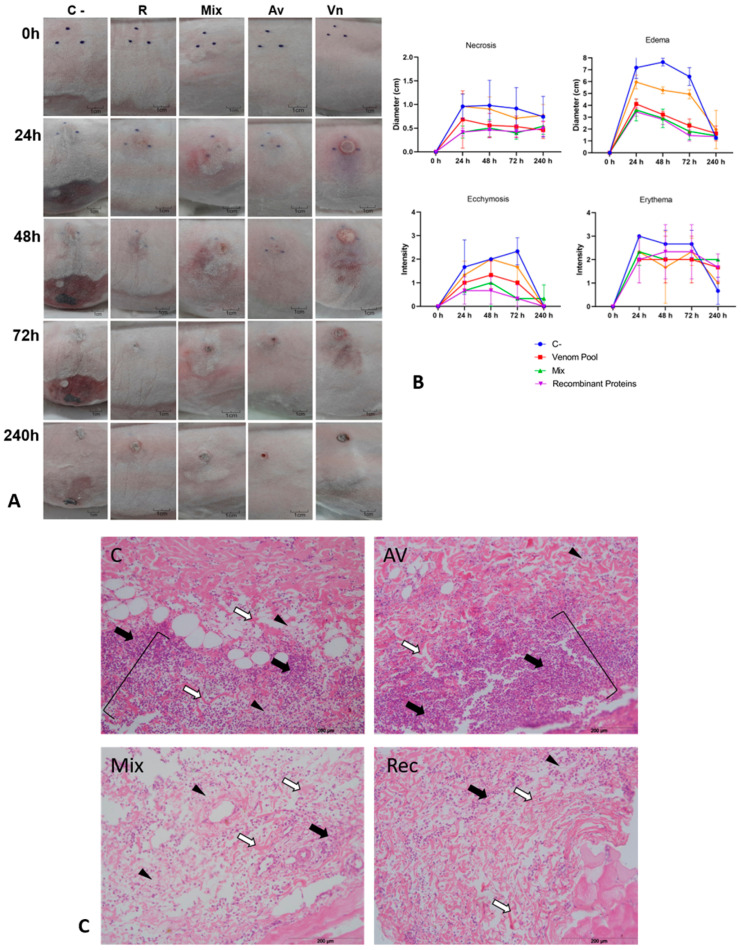
Neutralization of cutaneous loxoscelism triggered by 2 MND of venom tested with pre-incubation. Two MND (6.10 µg) of *L. intermedia* venom previously incubated with each serum were injected (I.D.) in the back skin of rabbits (groups of five animals). (**A**) Macroscopic aspect of injected area after the indicated time courses. C-: C serum. R: REC serum. M: MIX serum. Av: AV serum. Vn: 2 MND of *L. intermedia* venom in the absence of serum. Photographs show a representative response to the animals. Black dots marked on the skin surround the local injection. (**B**) Skin necrosis, edema, ecchymosis, and erythema were checked after various time courses (X axis) following injections of 2 MND of venom pre-incubated with each serum. The intensity of such signs (Y axis) was classified from 1 to 4 according to the intensity, in which “1” represents a less intense reaction and “4” indicates a more intense sign (adapted from [35]). Each point represents the average intensities of each group measured at the indicated times ± SD. Antivenom: AV serum. Recombinant proteins: REC serum; Mix: MIX serum. C-: C serum. *L. intermedia* venom: 2 MND of *L. intermedia* venom in the absence of serum. For necrosis, edema, and ecchymosis, results of the groups AV, MIX, and REC were statistically different from C (ANOVA, *p* < 0.005 for necrosis and ecchymosis, *p* < 0.0001 for edema). No significant difference was found among groups when erythema was analyzed. In all analyses, no significant difference was found among AV, MIX, and REC. (**C**) Histopathological analyses of rabbit skin after 48 h of injection with the incubation mixture of 2 MND of venom plus each serum. Tissue sections were stained with hematoxylin and eosin (HE). The images presented are representative of 2 analyzed sections and show the lower layer of the dermis, which usually concentrates the most evident changes in this tissue after *Loxosceles intermedia* venom I.D. injection. Scale bars are indicated in the figures. C: C serum. AV: AV serum. Mix: MIX serum. REC: REC serum. Closed arrows point out to leukocytes infiltrating the connective tissue. Square brackets in the upper panels delimit a massive infiltration of inflammatory cells that appear as a palisade of polymorphonuclear leukocytes. Disorganization of collagen bundles (open arrows) and fibrin deposition (arrowheads) are also present.

**Figure 7:**
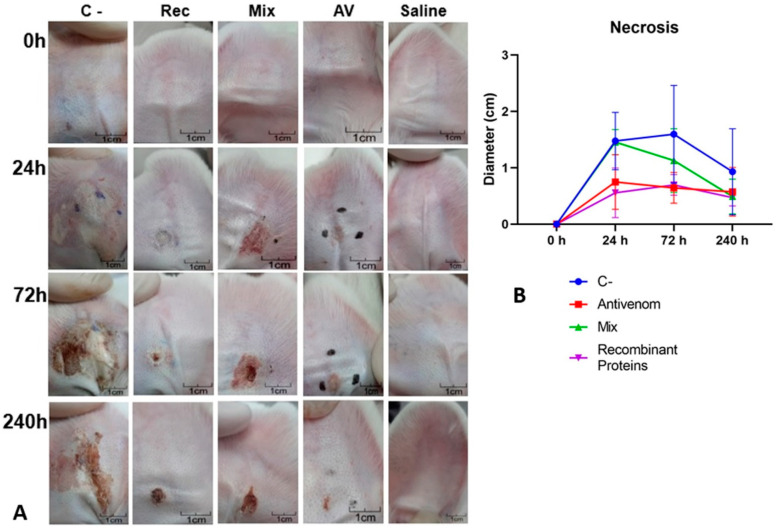
Neutralization of cutaneous loxoscelism in the ear pinna of rabbits. One MND (3.05 µg) of *L. intermedia* venom was injected (I.D.) in one ear of rabbits (groups of five animals). Immediately after venom injection, 1 mL of the tested serum was administered via the marginal ear vein of the opposite ear. (**A**) Macroscopic aspect of the area injected with venom after the indicated time courses. C-: C serum. Rec: REC serum. Mix: MIX serum. Av: AV serum. Saline: Inoculation of saline instead of venom. Photographs show a representative response to the animals. Black dots marked on the skin surround the local injection. (**B**) Skin necrosis was checked after various time courses (X axis) after injections of 1 MND, followed by inoculation of the tested sera. The intensity of necrosis (Y axis) was classified from 1 to 4 according to the intensity, in which “1” represents a less intense reaction and “4” indicates a more intense sign (adapted from [35]). Each point represents the average intensities of each group measured at the indicated times. Antivenom: AV serum. Recombinant proteins: REC serum; Mix: MIX serum. C-: C serum. Results of the groups AV (ANOVA, *p* = 0.0005) and REC (ANOVA, *p* = 0.0017) were statistically different from C at 72 h. No significant differences were found when MIX group was compared to C and neither among AV, REC, and MIX at 72 h.

In order to verify if the produced sera are able to neutralize the main local signs of cutaneous loxoscelism, two neutralization assays were performed. The first one examined the effects of a *Loxosceles* venom pre-incubated with the produced sera in rabbits’ dorsum skin (Figure 5 and Figure 6). The second assay evaluated if *Loxosceles* venom inoculated intradermally in the ear pinna of rabbits could be neutralized by the produced sera injected intravenously (Figure 7).

In the pre-incubation experiment performed in the skin of rabbits, two different amounts of *L. intermedia* venom were tested (1 MND and 2 MND) and analyzed separately (Figure 5 and Figure 6). When the development of dermonecrosis was examined, all specific sera significantly decreased the necrosis triggered by 1 or 2 MND of venom when compared to the negative control serum (C-). After 72 h, the findings for the 1 MND treatment showed that the MIX serum was able to reduce the development of the dermonecrotic lesion by 48.75% when compared to the C- serum. Additionally, AV and REC serum reduced the dermonecrotic reaction by 58.56% and 63.39%, respectively, when compared to the negative control (Figure 5A,B). The decrease in the development of dermonecrotic lesions with 2 MND of venom by the specific sera was 41.81% for the AV serum, 53.6% for the REC serum and 56.33% for the MIX serum (Figure 6A,B). Edema formation was also evaluated in this experiment. The measurements showed that all specific sera groups presented a significant minor edema formation when compared to the C- group for both 1 and 2 MND treatments (Figure 5B and Figure 6B). In the groups that were tested with 1 MND of *L. intermedia* venom, AV serum prevented the edema formation in 81.03% when compared to C- serum, which was similar to the reductions observed for the MIX and REC groups: 81.39% and 82.5%, respectively (Figure 5B). Although the prevention of edema by the specific sera was lower when 2 MND of venom was tested, it was still significant: measurements indicate a reduction of 64.33% by AV serum, 71.96% by MIX serum and 77.41% by REC serum (Figure 6B). No ecchymosis was observed in the groups that were tested with AV, REC, or MIX sera and 1 MND of venom (Figure 5A,B). Conversely, the specific sera were not as effective in the prevention of ecchymosis triggered by 2 MND of venom, although these groups still present significant differences with the C- group (Figure 6B). After statistical analyses regarding the prevention of dermonecrosis, edema, and ecchymosis, no significant differences were found among the neutralization indices of AV, MIX, and REC sera for both 1 and 2 MND treatments. Finally, none of the sera proved to be efficient in reducing the onset of erythema (Figure 5A,B, and Figure 6A,B).

Histopathological analyses of rabbits’ skin submitted to the pre-incubation experiment with 2 MND of *L. intermedia* venom have shown that some usual injuries were observed when the venom was inoculated, such as collagen fibers disorganization and the presence of fibrin in the connective tissue. These findings were present in all samples, including those in which a specific serum was pre-incubated with the venom (Figure 6C). However, it is possible to observe that the accumulation of polymorphonuclear cells was less pronounced in the MIX and REC groups (Figure 6C—lower panels) when compared to the massive infiltration of inflammatory cells that appear as a palisade of polymorphonuclear cells in the lower layer of the dermis seen for the AV and C- treatments (Figure 6C—upper panels). 

**Figure 8:**
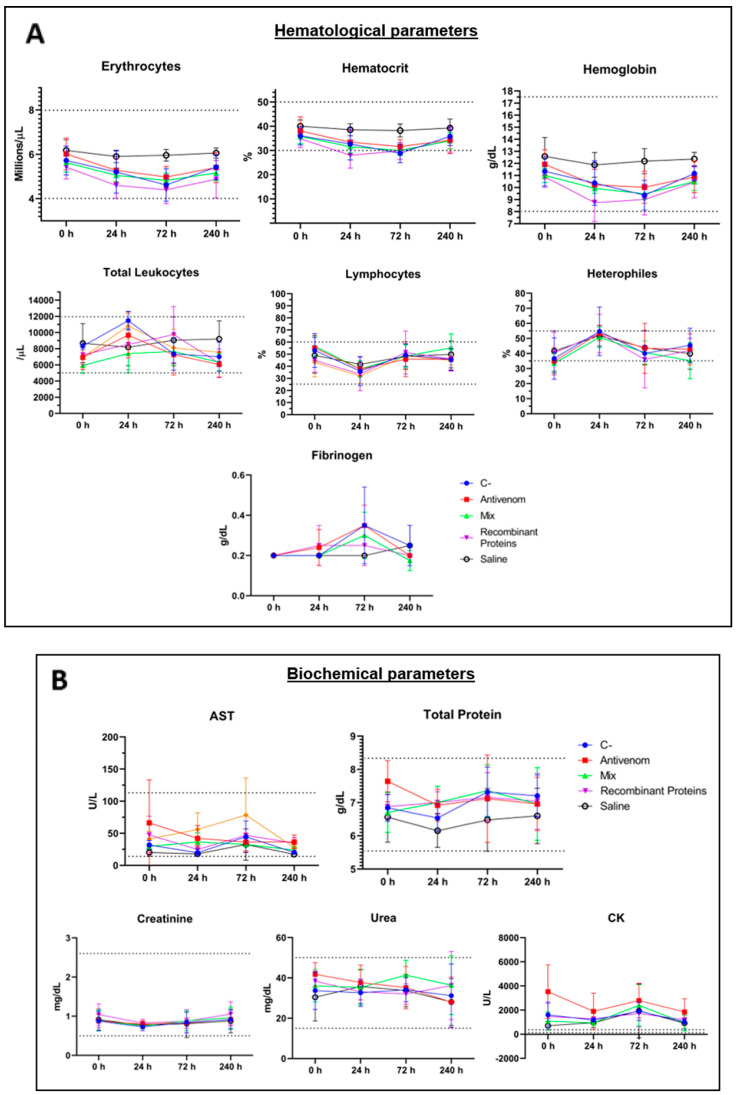
Monitoring of blood parameters of rabbits submitted to the neutralization test in the ear pinna. Hematological (**A**) and biochemical (**B**) parameters were verified from blood samples collected in different time courses (X axis). C-: C serum. Antivenom: AV serum. Mix: MIX serum. Rec: REC serum. Saline: Inoculation of saline instead of venom. AST: aspartate aminotransferase. CK: muscular creatine kinase. Lymphocytes: relative lymphocyte count. Heterophiles: relative heterophile count. The dashed lines indicate upper and lower reference values. For all parameters analyzed, no significant differences were found among the control groups (C and Saline) and AV, REC, and MIX groups.

The neutralization experiment performed in the ear pinna of rabbits aimed to evaluate the neutralization capacity of the produced sera by performing a required step in the quality control of the traditional serum currently used in the treatment of the victims. As demonstrated in Figure 7A,B, AV and REC sera were efficient in neutralizing the dermonecrotic effect of *L. intermedia* venom (1 MND) when compared to the neutralization observed for the C- group. However, this neutralization was not observed when the MIX serum was tested. The development of dermonecrotic lesions evaluated 72 h post-injection was reduced by 56.42% by REC serum, 59.74% by the AV serum, and 29.21% by the MIX serum (Figure 7A,B). To verify if serum administration triggered toxic effects in the tested animals, blood samples were collected after 0, 24, 72, and 240 h post-inoculation. The graphs presented in Figure 8 demonstrate that the animals administered with any of the sera tested did not present hematological (Figure 8A) or biochemical (Figure 8B) indices exceeding the reference values determined for rabbits. In addition, no significant differences in parameters were detected when the specific sera were compared to each other, except for a slight difference in red blood cell count for the AV and REC groups (*p* = 0.0254).

## 4. Discussion

The current approach based on serum to treat victims bitten by spiders of the genus *Loxosceles* is controversial from the efficacy standpoint since it has been reported that the serum was not able to prevent the development of signs of loxoscelism. Additionally, the current serum therapy is often the target of criticism due to technical aspects related to the production, which involves animal suffering used to raise the serum, the risk brought to personnel during the extraction of venom to be used in the immunization, and the inconvenient process of maintaining many spiders at the laboratory to extract this venom. The new approach based on mutated recombinant PLDs herein presented is promising since it overcomes all the issues mentioned above. SDS-PAGE analysis of the whole venom of *Loxosceles* spiders indicates the presence of two major groups of toxins in approximately equivalent amounts [14]. These two groups are related to the PLDs (31–35 kDa) and the insecticide peptides of the knottin family (6–10 kDa). The knottin peptides are innocuous to the victims since they are specific to insects that are predated by spiders in the natural environment. Thus, when the whole venom is used to raise the therapeutic serum, the immune system of the victims directs part of its machinery to elicit antibodies against molecules that are not detrimental. This may account for the low efficacy of the current serum therapy. The serum that this work studied has the advantage of being focused on the PLDs, which are the class of toxins that are responsible for the major signs triggered by the envenoming. This approach may be clinically more effective since the antibodies raised were all directed against the PLDs. Furthermore, because the mutated PLDs are engineered to stimulate the production of neutralizing antibodies but not induce harm in the immunized animal, the problem regarding the injuries in those animals is solved. Finally, since the mutated PLDs are produced by means of molecular biology techniques, there will be no need to collect and keep spiders, as well as no need to perform hazardous venom extraction.

The techniques for producing mutated and wild-type recombinant proteins (LgRecD32A-E34A, LiRecW230A, LlRecH12A-H47A, and LiRecDT1) analyzed in this work have been successfully used by several authors over the last few years [16,22,24,25]. Native amino acids were replaced by a residue of alanine (D32A-E34A, W230A e H12A-H47A), a non-polar amino acid capable of changing the functionality of wild-type proteins. The residue of alanine incorporated does not alter PLDs’ native conformation, which was proved by circular dichroism and docking analysis [22,24,25].

Through in vitro analysis, it was observed that the wild-type recombinant PLD (LiRecDT1) degraded sphingomyelin, while the mutated toxins did not show any significant activity. Likewise, the in vivo assay showed that LiRecDT1 caused extensive dermonecrosis on rabbit skin, whereas the mutated proteins did not lead to the development of lesions, except for LlRecDTH12A-H47A, which caused a slight residual inflammatory reaction around 24 h after injection. Such results were previously reproduced by several authors [16,22,24,25] and confirm the success of the amino acid replacements in the mutated proteins applied herein in further experiments, preventing their biochemical and functional activities.

With the evidence that the mutated proteins do not have biochemical and functional activities but have their structural characteristics preserved, different groups of rabbits were immunized in order to produce sera to be evaluated. Studies available in the literature that aimed at the clinical follow-up of animals producing antivenom serum are scarce [37,38,39]. Therefore, a greater knowledge of this subject becomes essential for the improvement of the processes. Thus, in this work, we produced two hyperimmune sera using recombinant PLDs from *Loxosceles* spider venoms, which were evaluated for their efficiency, as well as their antigens toxicity during the protocols for obtaining the sera. From the results herein generated, it is believed that the greater intensities of erythema and ecchymosis during the immunization protocols using recombinant mutated proteins alone may be directly related to the large mass of antigens applied to the animals, plus the adverse effects of adjuvant [40]. On the other hand, the signs of erythema, ecchymosis, and the increase in serum fibrinogen after the first immunization observed in the animals that received crude venom or a mixture of crude venom and mutated PLDs (AV and MIX) can be explained by the action of the crude *Loxosceles* venom toxins administered to these animals, in addition to the adjuvant effects [27,41].

The hematocrit, erythrocyte count, and hemoglobin concentration in the sample of the tested animals were not altered, suggesting that there was no hemolysis caused by the toxins in the conditions used [42]. This finding is probably related to the low concentration of molecules that was gradually inoculated into the animals, not inducing systemic changes [19,43]. The differences between the lymphocytes and heterophils curves observed throughout the immunization protocols are coherent, considering the cellular dynamics of the immune response and the inflammatory profile triggered by some of the toxins present in the venoms [44].

In the analysis of serum protein concentrations, there was a significant difference between the MIX treatment and the negative control, which despite receiving only adjuvants in the composition of the immunogens, had a higher serum protein concentration. This fact may be related to the pro-inflammatory effects triggered by adjuvants and to an eventual greater susceptibility of individuals in this group to the adjuvant side effects [40].

Liver function was verified by measuring the concentration of the enzyme aspartate aminotransferase (AST) since the enzyme alanine aminotransferase (ALT), also commonly analyzed in other animals, has little tissue specificity in rabbits [45]. Despite the hepatotoxic effect of *Loxosceles* venom reported by [46], all groups had enzyme concentrations within the reference values for the animals tested [47], not indicating liver damage resulting from immunization processes under the conditions used.

Values above the reference levels for creatine kinase (CK) were found in all analyzed points, indicating lesions in skeletal muscle resulting from immunizations. This fact was also observed in immunizations in other species [37,48] and is directly correlated with the adverse effects of the use of oily adjuvants. These adjuvants can cause local reactions derived from the damage to cells and even granulomas at the site of inoculation or which arise from animal manipulations [40,49].

Based on the concentrations of urea and creatinine found and considering that the concentration of crude venom applied to the animals was small, it can be stated that the renal function was not affected during the immunization processes, although the nephrotoxic capacity of Brown spider venoms has already been demonstrated both experimentally [16] and clinically from injured patients [9].

There are reports in the literature indicating that the application of large doses of antigens can lead to greater production of antibodies, showing a possible relationship between the two variables [50,51,52]. However, this premise cannot be taken as a rule. In the ELISA graph (Figure 4A), it is observed that the sera obtained from the MIX and AV groups presented similar curves, although the concentration of venom of the three species of Brown spiders injected in the animals belonging to the MIX group was up to six times lower than that used for the AV group. On the other hand, the serum obtained by using only recombinant proteins showed a significant difference when compared to the others. In that case, the animals received 2.5 μg more of mutated recombinant proteins than the MIX group. This probably increased the stimulus for clonal expansion of B lymphocytes against the antigens [44] since they are in greater concentration and have smaller heterogeneity when compared to the MIX group, which has three venoms and three mutated proteins.

In the Western blotting assays, it was verified that sera obtained from MIX and REC groups presented more intense reactions on the 25–35 kDa region. This fact may be related to the immunizations since these groups received mutated phospholipases in a large concentration, causing the production of antibodies to be greater than that of the AV group, whose animals received smaller concentrations of phospholipases of the three species of Brown spiders [50,51,52]. The recognition of bands with low molecular weight is probably due to the presence of degraded phospholipases in the venom analyzed. Serum cross-reactivity, as observed in this trial, has been described by several authors using different anti-*Loxosceles* sera and venoms [30,53]. This characteristic is essential and desirable for the development of a product with great capability of neutralization and recognition of venoms from a large number of species.

In the in vivo incubation test for serums and toxins, after 72 h of inoculation of the mixture, the sera produced from immunizations with the REC treatment had a superior capability to reduce edema and dermonecrotic lesions (except for the group MIX when 1 MND of venom was used), when compared to serum raised from the AV preparation. The residual reactions observed are due to the action of non-neutralized toxins, possibly other than PLDs. In the neutralization assay similar to that conducted here, Leal [54] verified that individual sera obtained using recombinant phospholipases D (recLoxtox s1A) or (recLoxtox s11A), and the mixture of both sera, significantly reduced the appearance of dermonecrotic lesions in rabbits exposed to the venom. These findings were similar to those described herein and suggest that the presence of specific antibodies against dermonecrotic toxins (PLDs) is essential and plays a role in the positive evolution of the clinical picture of Loxoscelism. All sera produced in this work were efficient in reducing the swollen areas after venom exposure. As reported by Leal [54] that obtained a significant reduction in the diameter of edema formed after inoculation of crude venom, similar data were acquired herein from animals immunized with the recombinant PLDs. These results suggest that specific antibodies for the neutralization of certain PLD epitopes can contribute to the reduction of edema formation.

Recombinant proteins previously described, such as LgRec1, LgRecDT1, and *Loxosceles gaucho* crude venom itself, have the capacity to form ecchymosis in rabbits [24,55,56]. Thus, the neutralization of this clinical sign, especially for the groups that received 1MND of venom, was consistent with the hypothesis of reduced disruption of the vessels’ wall through the neutralization of venom toxins by antibodies from this second-generation serum using recombinant PLDs.

Erythema is also a common sign observed in experimental or clinical Loxoscelism [24,55]. However, in the work described here, none of the sera produced were efficient in preventing the erythema following venom exposure. Knowing that the signs observed in Loxoscelism are due to the synergism action of the components of the venoms [54], it is suggested that other toxins such as metalloproteases and hyaluronidases, act as spreading factors [10], as well as pro-inflammatory agents that may be involved with the formation of erythema and ecchymosis.

Currently, during the production of commercial anti-loxoscelic serum, the plasma of immunized horses is tested through neutralizing assays performed in the ear of rabbits in order to determine which horses produced a minimum amount of antibodies capable of neutralizing the action of the venom. The same protocol is used in evaluating the final product. The test consists of an intradermal application of one MND of *Loxosceles intermedia* crude venom in the inner portion of the ear of rabbits (ear pinna). In the opposite ear, the plasma or serum is injected intravenously. The evaluation of the effectiveness of the serum takes place 72 h after its application (Official Protocol for Anti-*Loxosceles* serum production, CPPI, Brazil, 2014, 2017). This test was also carried out with sera produced in this work to assess the quality of the sera produced (quality control step). There was no difference when the produced sera were compared 72 h after the inoculation of the preparations. The reduction of lesions was less significant when compared to the incubation assay performed. It is believed that this is due to at least one of the following factors: i) in the incubation assay, longer exposure times of antibodies and antigens allow better recognition of epitopes and a greater number of molecular bindings. In the ear serum neutralization test, the sera were administered in the opposite ear that received the venom, causing the molecules to travel a long way through blood vessels until they found the venom antigens; ii) mechanism of action of the venom toxins. Although the clinical signs of Loxoscelism appear a few hours after the bite, the venom, when in contact with its substrates, acts promptly, and it is expected that the activation of leukocytes occurs within 4 h [27,57]. This fact can be commonly observed in the quality control assays of the serum currently produced. The hypothesis that justifies the lower efficacy of the serum obtained for the group MIX in this trial derives from the data reported by Figueiredo [36], who proposed that genetic and individual conditions of each animal used to produce antibodies can generate different sera since they do not come from isogenic strains. Such events are also observed in the production of commercial serum, which can show differences regarding the potency for each batch due to variations in the production of antibodies from the same animal over a period.

The anti-*Loxosceles* serum, if injected in the first six hours after the accident, can reduce up to 90% of the dermonecrotic lesion [27]. It is believed that this difference with the sera produced on this project is due to the fact that the processed serum tested by [27] has a very high concentration of antibodies, being able to neutralize at least 15 times the MND. In addition, anti-*Loxosceles* serum-producing horses are immunized more frequently over the course of a year (about four immunization cycles, whereas the animals tested here have gone through only one), which may influence a greater production of antibodies.

Considering that 1MND of *L. intermedia* venom is not capable of causing serious alterations in rabbits, as evidenced by the negative control group, any possible alteration in the test results would indicate serum toxicity. The animals that received the sera did not show non-standard clinical indices for the species [47], except for the enzyme creatine kinase (CK), used as a marker for muscle damage. It is considered that high concentrations of CK may be related to the handling of animals, resulting in stress and injuries caused by containment, application of antigens, application of serums, and blood collections.

## 5. Conclusions

After the experimental protocol carried out in this work, we can suggest that sera produced from immunizations with mutated recombinant proteins without biological activity proved to be as efficient as the traditional anti-*Loxosceles* serum currently used, obtained from immunizations with the crude venoms of the three species of Brown spiders of major medical importance in Brazil and South America *L. gaucho, L. intermedia* and *L. laeta.* The results observed and gathered herein indicated no significant differences between the serum based on mutated PLDs and the serum obtained using crude venom regarding the development of dermonecrotic lesions and edema in the immunized animals. The results presented show the potential use of these antigens to produce the second-generation anti-*Loxosceles* serum, also because the use of mutated PLDs minimizes the need for capture and maintenance of specimens of *Loxosceles spp* ex situ for the venom extraction step, which is characterized by being laborious, low yield, requiring thousands of spiders to complete the equine immunization cycles, and finally, the danger of spider bites during manipulation. This step could be replaced completely or partially by the production of recombinant proteins in the laboratory.

The results presented here, although suggestive and technically robust, need additional evidence. Other important data should be reported in future studies aiming a change in the current production model to obtain anti-*Loxosceles* serum, such as the verification of the minimum concentration of antigens capable of stimulating an adequate immune response (cost–benefit), evaluation of immunization in horses instead of rabbits and the assessment of suitable hyperimmune plasma processing methodologies. Nevertheless, the data presented here highlight that mutated recombinant PLDs are promising tools to produce anti-*Loxosceles* serum with higher efficacy and other operational advantages over the traditional approach.

## Figures and Tables

**Table 1:** Protocol to produce sera.

Group Name	Antigen	1st Injection	2nd Injection	3rd Injection	4th Injection
AV	Venom pool *	7.5 µg of pool + CFA	10 µg of pool + IFA	10 µg of pool + IFA	15 µg of pool + IFA
REC	Recombinant proteins’ pool **	100 µg of pool + CFA	200 µg of pool + IFA	400 µg of pool + IFA	800 µg of pool + IFA
MIX	Venom pool * + Recombinant proteins’ pool **	2.5 µg of venom pool + 97.5 µg of recombinant protein’s pool + CFA	2.5 µg of venom pool + 197.5 µg of recombinant protein’s pool + IFA	2.5 µg of venom pool + 397.5 µg of recombinant protein’s pool + IFA	2.5 µg of venom pool + 797.5 µg of recombinant protein’s pool + IFA
C	Negative control	0.85% saline + CFA	0.85% saline + IFA	0.85% saline + IFA	0.85% saline + IFA

* Venoms of *L. gaucho*, *L. intermedia* and *L. laeta*—1:1:1. ** Mutated recombinant proteins LiRecDT1 W230A, LlRecDT1 H12A-H47A and LgRecDT1 E32A-D34A—1:1:1. CFA: Complete Freund’s Adjuvant. IFA: Incomplete Freund’s Adjuvant.

## Data Availability

Data are contained within the article.
